# Sleep Disturbances and Cognition, Behavior, and Brain Structure in Children With mTBI

**DOI:** 10.1001/jamanetworkopen.2026.0229

**Published:** 2026-03-10

**Authors:** Anja K. Betz, Hanneke S. R. MacLaren, Alberto G. Villagran Asiares, Luisa S. Schuhmacher, Inga K. Koerte

**Affiliations:** 1 cBRAIN, Department of Child and Adolescent Psychiatry, Psychosomatics and Psychotherapy, Ludwig-Maximilians-Universität, Munich, Germany; 2 Psychiatry Neuroimaging Laboratory, Department of Psychiatry, Mass General Brigham, Harvard Medical School, Boston, Massachusetts; 3 Graduate School of Systemic Neurosciences, Ludwig-Maximilians-Universität, Munich, Germany; 4 International Max Planck Research School for Translational Psychiatry, Munich, Germany; 5 German Center for Child and Adolescent Health (DZKJ), Partner site Munich, Munich, Germany

## Abstract

**Importance:**

Sleep disturbances are increasingly recognized as a modifiable factor associated with poor outcomes after mild traumatic brain injury (mTBI) in adults, yet their prevalence, mechanisms, and clinical impact in children remain largely unknown. Identifying whether sleep problems contribute to behavioral, cognitive, and neurobiological outcomes after pediatric mTBI could inform targeted interventions.

**Objective:**

To determine the prevalence of sleep disturbances after pediatric mTBI and to assess associations with behavioral symptoms, cognition, and brain structure.

**Design, Setting, and Participants:**

This multisite, population-based cohort study is a secondary analysis of data collected between September 2016 and January 2020 as part of the Adolescent Brain Cognitive Development Study 5.0 release. Participants were children who sustained an mTBI between baseline (age 9-10 years) and 2-year follow-up (age 11-12 years). Children with mTBI were propensity score–matched on the basis of age, sex, study site, self-reported race, and total family income to typically developing children (TDC) and orthopedic injury (OI) controls. Data were analyzed September 2024 to June 2025.

**Exposure:**

mTBI between baseline and follow-up.

**Main Outcomes and Measures:**

The primary outcomes were parent-reported sleep disturbances (categorized as new onset, chronic, improving, or none), behavioral problems, and cognitive performance. Magnetic resonance imaging was used to examine cortical thickness, cortical volume, and white matter microstructure. Analyses used linear regressions and linear mixed models, controlling for baseline when appropriate.

**Results:**

Of 573 children, there were 191 children with mTBI (mean [SD] age, 12.03 [0.6] years; 112 [58.6%] male). Children with mTBI were more likely than controls to develop new clinical sleep disturbances (29 children with mTBI [15.2%] vs 22 of 191 children [11.5%] in the TDC group and 19 of 191 children [9.9%] in the OI group) and had higher rates of chronic sleep disturbances (41 children [21.5%] with mTBI vs 25 children [13.1%] in the TDC group and 25 children [13.1%] in the OI group). Total sleep disturbance scores were significantly elevated for children with mTBI compared with the TDC group (β, −0.27; 95% CI, −0.45 to −0.10), but not the OI group (β, −0.12; 95% CI, −0.29 to 0.05). Higher behavioral symptoms were found compared with the TDC group (β, −0.30; 95% CI, −0.45 to −0.16) and were associated with sleep problems, especially newly emerging disturbances, after mTBI. Children in the OI group exhibited greater cortical thickness (β, 0.18; 95% CI, 0.06 to 0.30) and volume (β, 0.06; 95% CI, 0.01 to 0.11) than children with mTBI, with both being positively associated with sleep disturbances in children with mTBI. No group differences were found in cognition or white matter microstructure or associations with sleep.

**Conclusions and Relevance:**

In this cohort study, sleep disturbances were more common among children with mTBI than among TDC. In particular, newly developing sleep problems appeared to be a potential key pathway to behavioral problems and a promising interventional target. Cognitive and structural brain outcomes were not significantly associated with sleep disturbances in this cohort.

## Key Points

**Question:**

Is pediatric mild traumatic brain injury (mTBI) associated with sleep disturbances and increased risk of poor behavioral, cognitive, and brain structural outcomes compared with typically developing children and orthopedic injury controls?

**Findings:**

In this cohort study of 573 children, the 191 children with mTBI had significantly higher total sleep disturbance scores than the 191 typically developing children, but not compared with the 191 children in the orthopedic injury control group. Of all examined outcomes, behavioral problems were most consistently associated with sleep disturbances, especially those developing only after injury.

**Meaning:**

These findings suggest that newly emerging sleep disturbances after mTBI may be a modifiable risk factor for behavioral problems and a potential target for early intervention in pediatric brain injury recovery.

## Teaser

This cohort study investigates the associations of pediatric mild traumatic brain injury (mTBI) with increased risks of behavioral, cognitive, and brain structural outcomes compared with typically developing children and an orthopedic injury control group.

## Introduction

Mild traumatic brain injury (mTBI), or concussion, is a common neurological condition in children.^1^ Approximately 30% of cases^2^ result in persistent physical, cognitive, and emotional symptoms,^3^ which are often accompanied by brain structural changes, including decreased white matter volume^4^ and cortical thinning.^5^ It remains unclear why symptoms persist in only a subset of children, although risk factors like sex, age, injury characteristics, and preinjury psychiatric conditions have been identified. However, certain postinjury symptoms, such as sleep disturbances, may also contribute to a prolonged recovery.

Sleep disturbances impact 53% to 92% of adult patients with mTBI^6,7^ and are recognized as both an independent symptom domain and a risk factor for poor outcomes.^3^ They have been linked to anxiety and depression,^8^ slower cognitive recovery,^9^ and reduced brain volume.^10^ Emerging evidence in children suggests that approximately 28% experience posttraumatic sleep problems.^11^ However, pediatric studies that examine sleep in association with other outcomes are rare, and most do not include control groups. In patient-only cohorts, poor sleep has been associated with worse cognitive outcomes,^12^ increased postconcussive symptoms,^13^ and lower neurite density.^14^ Longer and more efficient sleep, in turn, was associated with an earlier return to school.^15^ Comparisons across injury types are, however, essential for isolating mTBI-specific impacts. Some studies showed few differences between mTBI and orthopedically injured (OI) controls,^16^ while both groups experienced worse sleep than typically developing children (TDC).^17^ Associations of sleep with behavioral problems^16,17^ and postconcussive symptoms^18^ were also not exclusive to mTBI. Finally, in TDC, poor sleep is associated with higher behavioral and emotional problems,^19,20^ lower white matter fractional anisotropy (FA),^21^ and lower cortical volume and thickness.^22^ These findings highlight both the importance of sleep in multiple neurodevelopmental domains and the need for incorporating preinjury characteristics and multiple control groups in pediatric mTBI research.

This study investigates the prevalence and role of sleep disturbances in children with mTBI compared with 2 propensity score–matched TDC and OI control groups. Using data from the Adolescent Brain and Cognitive Development (ABCD) study, we focus on children who sustained an mTBI between baseline and the 2-year follow-up (collected between September 2016 and January 2020). We hypothesize that children with mTBI will have higher sleep problems than TDC and, to a lesser extent, OI controls. We examine sleep disturbances and their associations with behavioral problems, cognitive functioning, cortical thickness, cortical volume, and white matter microstructure, all measured at the 2-year follow-up appointment after the injury. On the basis of the literature on adults with mTBI and healthy children, each of these additional outcomes is expected to show an association with sleep. We aim to contribute to the characterization of sleep problems after pediatric mTBI by leveraging a large, longitudinal, population-based dataset that allows us to take both preinjury factors and postinjury outcome development into account. By including 2 matched control groups, we also address inconsistencies in the previous literature and attempt to isolate the impacts of a brain injury.

## Methods

### Sample

This cohort study uses data from release 5.0 of the US-based, longitudinal, multisite ABCD study.^23,24^ Institutional review board approval was obtained by the University of California, San Diego. Parents and caregivers provided written informed consent, while children provided written assent. This study followed the Strengthening the Reporting of Observational Studies in Epidemiology (STROBE) reporting guidelines. Children were enrolled at ages 9 to 10 years for the baseline appointment and were followed up annually, with a magnetic resonance imaging (MRI) scan every 2 years. This study focuses on the baseline (ages 9-10 years) and 2-year follow-up assessments (ages 11-12 years). At each of the 2 time points, parents completed the ABCD Parent Ohio State Traumatic Brain Injury Screen–Short Modified. ^25^ Children were classified as having sustained an mTBI if they experienced loss of consciousness, memory loss, or feeling dazed or confused after a suitable event (eg, a fall). For the mTBI group, we included only children who had no history of head injury at baseline and whose parents reported a new injury at either the 1-year or 2-year follow-up. This approach allows the use of baseline measurements as preinjury reference values.

Children with mTBI were propensity score–matched with a 1:1 ratio to TDC without a history of injury and OI controls with broken bones within the same time frame. Matching was conducted with the Match.it package^26^ in R version 4.1.2 (R Project for Statistical Computing) on the basis of age, sex, study site, self-reported race, and total family income. All matching variables were included as covariates in subsequent analyses to account for within-group influences. Reported races included American Indian and Alaska Native, Black, Native Hawaiian and Pacific Islander, White, and Other. While the mTBI sample was predominantly White, the matching was meant to ensure that the control groups also retained similar percentages of additional racial groups. A similar rationale applies to the matching on family income—while more families from the mTBI group were relatively wealthy, we aimed to retain the demographic diversity that was available to maximize generalizability. Details on sample selection can be found in eAppendix 1 in Supplement 1.

### Measures

Sleep problems were assessed using the parent-reported Sleep Disturbance Scale for Children (SDSC),^27,28^ which includes 6 subscales: disorders of initiating and maintaining sleep, sleep breathing disorders, disorders of arousal, sleep-wake transition disorders, disorders of excessive somnolence, and sleep hyperhidrosis. Our main analyses focused on the continuous total score, with exploratory post hoc analyses of the subscales. Additionally, a total score greater than 39 indicated clinically relevant sleep problems^27^ and was used to categorize children into 4 groups: normal (no clinical sleep problems), chronic (persistent clinical sleep problems), improving (clinical sleep problems at baseline, not at follow-up), and new onset (no clinical sleep problems at baseline, but at follow-up).

Behavioral problems were assessed using the Child Behavior Check List,^29^ focusing on the total problems score. To avoid any confounding effects, we recalculated the total score excluding all sleep-related items.

Cognitive functioning was assessed with the National Institutes of Health Toolbox.^30^ We created a substitute composite score from the mean of the 3 age-corrected standard scores available for both time points: the oral reading recognition (language), pattern comparison (processing speed), and the flanker inhibitory control and attention (executive functioning) tests.

MRI was performed by the ABCD study team at each time point according to a standardized protocol.^31^ Quality control and preprocessing had been completed centrally.^32^ Given the lack of consistent findings in previous research, we examined whole-brain metrics: cortical thickness (millimeters), cortical volume (×10^5^ mm^3^), and FA (unitless). To reduce scanner-related variability across sites,^33^ we applied ComBat^34^ harmonization.

### Statistical Analysis

#### Main Analysis

Data were analyzed in R version 4.1.2 (R Project for Statistical Computing).^36^ To improve the robustness of our findings, all our analyses were bootstrapped with replacement from matched sets in 1000 iterations. Statistical inference was thereby based on bootstrapped 95% CIs of the estimates. Outcome variables were *z* scored relative to the sample in each bootstrapping iteration. Age at baseline, sex, study site, race, and total family income were consistently used as covariates. Linear regressions were used unless otherwise specified and controlled for the respective scores at baseline.

First, we calculated the proportions of all 4 categories of sleep development (normal, chronic, improving, and new onset) in the 3 groups (mTBI, TDC, and OI). Group differences in total sleep problems were compared via analysis of covariance. Second, group differences in behavior, cognition, cortical thickness, cortical volume, and FA were analyzed. Third, the association of sleep problems with these outcomes was assessed in the mTBI sample: To disentangle the temporal dynamics, the continuous SDSC score at follow-up was first used as an independent variable in regression models for each outcome, while controlling for the baseline value of the respective outcome. Then, we analyzed between-group and within-group differences for the different sleep trajectories by employing a linear mixed-effects model with a trajectory by time interaction. Finally, we performed exploratory analyses of group differences and associations on the subscales of the SDSC to identify specific sleep-related outcomes.

#### Sensitivity Analyses

We have included these sensitivity analyses, all of which are visualized in eAppendix 2 in Supplement 1. To examine generalizability, we repeated the main analyses excluding children with preinjury psychiatric diagnoses. Second, we investigated impacts stratified by sex. Finally, we investigated whether baseline sleep could estimate outcomes when moderated by group membership as was shown in a recent publication.^35^

## Results

### Demographics

**Figure 1.**
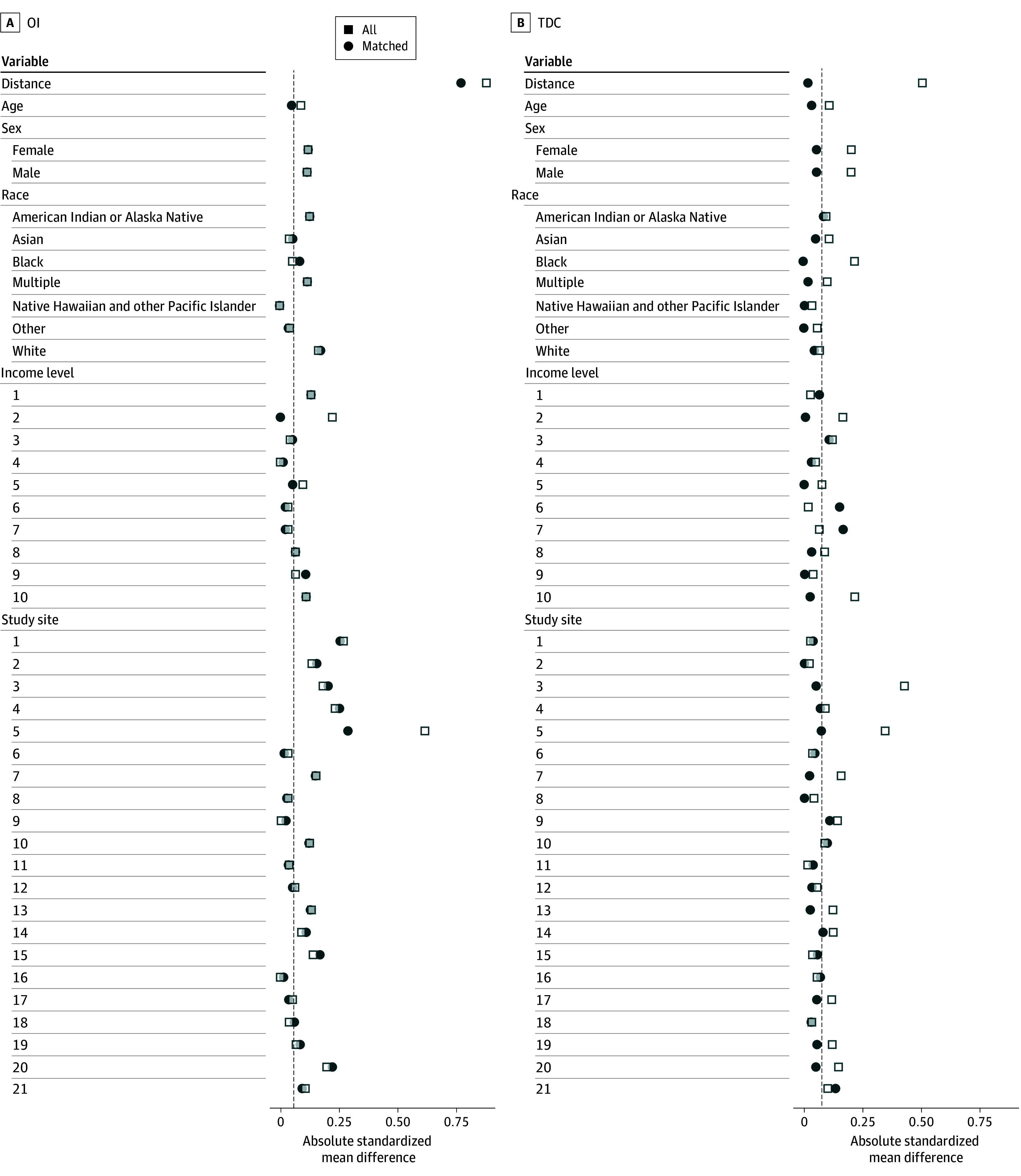
Love Plot of Standardized Mean Differences in Control Group Matching Visualization of standardized mean differences between children with mild traumatic brain injury and typically developing children (TDC) and orthopedic injury (OI) controls, respectively, before and after matching. Other race indicates participants who did not identify with any of the races otherwise listed.

A total of 213 children with mTBI were identified. One was excluded due to a change in study site between time points, and 21 were excluded due to missing demographic or sleep data, resulting in a final mTBI sample of 191 children (mean [SD] age, 12.03 [0.06] years; 112 [58.6%] male). Patients were matched to equally sized TDC and OI groups (total 573 children) (eFigure 1 in Supplement 1). Due to the limited number of eligible OI participants (216 children), study site remained significantly different between mTBI and OI after matching (eTable 1 in Supplement 1; Figure 1). Sample characteristics are presented in Table 1. For imaging analyses, participants for whom quality control failed were excluded from the respective analysis (9 children with mTBI, 9 children in the TDC group, and 8 children in the OI group for cortical thickness and volume, as well as 35 children with mTBI, 32 in the TDC group, and 27 in the OI group for white matter microstructure).

**Table 1.** Demographic Sample Characteristics of All Participants

Characteristic	Study group, No. (%)			*P* value^a^	
	mTBI (n = 191)	TDC (n = 191)	OI (n = 191)	mTBI vs TDC	mTBI vs OI
Age, mean (SD), y	12.03 (0.6)	12.05 (0.6)	12.00 (0.6)	.76	.63
Biological sex					
Male	112 (58.6)	107 (56.0)	101 (52.9)	.68	.30
Female	79 (41.4)	84 (44.0)	90 (47.1)		
Race					
American Indian and Alaska Native	3 (1.6)	1 (0.5)	0	.95	.33
Asian	2 (1.0)	1 (0.5)	3 (1.6)		
Black	13 (6.8)	13 (6.8)	9 (4.7)		
Multiple	30 (15.7)	29 (15.2)	22 (11.5)		
Native Hawaiian and other Pacific Islander	0	0	0		
Other^b^	5 (2.6)	5 (2.6)	4 (2.1)		
White	138 (72.3)	142 (74.3)	153 (80.1)		
Total family annual income, $					
<5000	5 (2.6)	7 (3.7)	1 (0.5)	.67	.68
5000-11 999	0	0	0		
12 000-15 999	2 (1.0)	4 (2.1)	3 (1.6)		
16 000-24 999	5 (2.6)	4 (2.1)	5 (2.7)		
25 000-34 999	7 (3.7)	7 (3.7)	9 (4.7)		
35 000-49 999	12 (6.3)	19 (9.9)	11 (5.8)		
50 000-74 999	22 (11.5)	12 (6.3)	18 (9.4)		
75 000-99 999	24 (12.6)	22 (11.5)	29 (15.2)		
100 000-199 999	70 (36.6)	70 (36.6)	80 (41.9)		
≥200 000	44 (23.0)	46 (24.1)	35 (18.3)		
Study site	NA	NA	NA	>.99	.01

Abbreviations: mTBI, mild traumatic brain injury; NA, not applicable; OI, orthopedic injury; TDC, typically developing children.

^a^ *P* value columns contain comparisons between mTBI and TDC or OI, respectively, calculated using *t* test (age), χ^2^ test (sex), and Fisher exact test (race, income, and site).

^b^ Other race indicates participants who did not identify with any of the races listed.

### Group Differences

#### Sleep Problems

**Figure 2.**
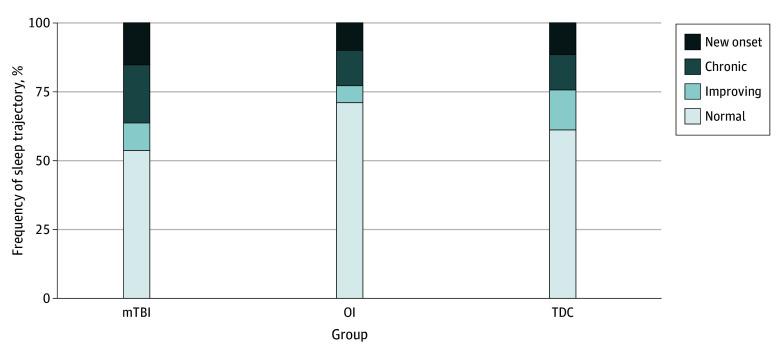
Bar Graph of Development of Sleep per Group Graph shows proportions of the different sleep trajectories in children with mild traumatic brain injury (mTBI), orthopedic injury (OI) controls, and typically developing children (TDC).

Descriptively, a higher proportion of children with mTBI developed new-onset clinical sleep problems (29 children [15.2%] with mTBI vs 22 children [11.5%] in the TDC group and 19 children [9.9%] in the OI group) and had higher rates of chronic sleep disturbances (41 children [21.5%] with mTBI vs 25 children [13.1%] in the TDC group and 25 children [13.1%] in the OI group). The OI group showed fewer improving cases, likely due to fewer baseline sleep problems (Figure 2). On a continuous scale, children with mTBI had higher sleep disturbance scores than the TDC group (β, −0.27; 95% CI, −0.45 to −0.10), but not the OI group (β, −0.12; 95% CI, −0.29 to 0.05). The control groups did not differ in terms of sleep disturbance score (β, −0.15; 95% CI, −0.31 to 0.02).

#### Group Differences in Outcome

Children with mTBI showed higher behavioral problems compared with the TDC group (β, −0.30; 95% CI, −0.45 to −0.16), as well as lower cortical thickness (β, 0.18; 95% CI, 0.06 to 0.30) and volume (β, 0.06; 95% CI, 0.01 to 0.11) compared with the OI group. No group differences were found regarding white matter microstructure and cognitive performance (Table 2; eFigure 2 in Supplement 1).

**Table 2.** Group Differences Between Mild Traumatic Brain Injury and Each Control Group, at the 2-Year Follow-Up, Controlling for Baseline

Outcome and group^a^	β, mean (95% CI)	*f*^2^, mean (95% CI)^b^
Cortical thickness (n = 434)		
TDC	0.08 (−0.05 to 0.21)	0.007 (0.000 to 0.028)
OI	0.18 (0.06 to 0.30)^c^	0.022 (0.002 to 0.056)^c^
Cortical volume (n = 434)		
TDC	0.03 (−0.03 to 0.08)	0.006 (0.000 to 0.025)
OI	0.06 (0.01 to 0.11)^c^	0.017 (0.001 to 0.047)^c^
Fractional anisotropy (n = 385)		
TDC	0.04 (−0.20 to 0.28)	0.004 (0.000 to 0.020)
OI	0.13 (−0.10 to 0.35)	0.007 (0.000 to 0.029)
Cognition (n = 462)		
TDC	0.01 (−0.17 to 0.21)	0.003 (0.000 to 0.015)
OI	0.09 (−0.09 to 0.29)	0.005 (0.000 to 0.020)
Behavior problems (n = 493)		
TDC	−0.30 (−0.45 to −0.16)^c^	0.034 (0.008 to 0.071)^c^
OI	−0.08 (−0.26 to 0.10)	0.005 (0.000 to 0.021)

Abbreviations: OI, orthopedic injury; TDC, typically developing children

^a^ Numbers in parentheses indicate the sample size across all 3 groups for each respective analysis.

^b^ An *f*^2^ of 0.02 or greater to less than 0.15 represents small effect sizes; 0.15 or greater to less than 0.35, medium effect sizes; 0.35 or greater, large effect sizes.

^c^ Denotes a 95% CI that does not include 0, indicative of statistical significance.

### Role of Sleep

#### Associations Between Sleep and Outcomes

**Figure 3.**
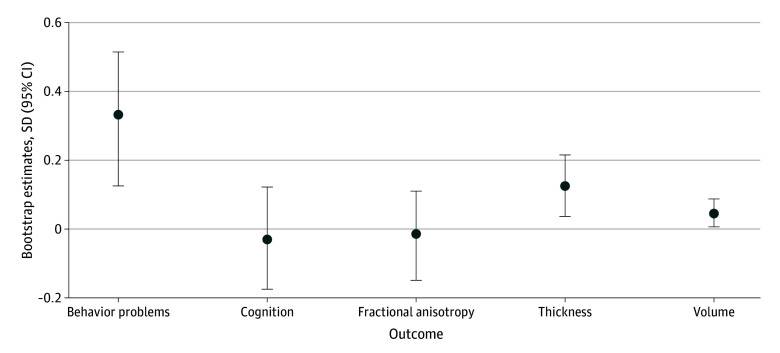
Dot-and-Whisker Plot of Associations of Sleep With Outcomes Graph shows associations of sleep at follow-up with outcomes in children with mild traumatic brain injury, controlled for baseline outcomes. Dots denote means, and error bars denote 95% CIs.

In the mTBI group, sleep problems were associated with behavioral problems, with cortical thickness, and with cortical volume. No associations were found for white matter microstructure or cognition (eTable 2 in Supplement 1; Figure 3). At the preinjury baseline, the chronic trajectory showed higher behavioral problems than both the new-onset and the normal trajectory, both of which did not have clinical sleep problems at that point. At the 2-year follow-up, behavioral problems in the new-onset trajectory had significantly increased and no longer differed from the children with chronic sleep problems, but both groups had higher behavioral problems than the children with consistently normal sleep (eFigure 3A and eTable 3 in Supplement 1). A different pattern could be seen in TDC, where the only change over time was an improvement of behavior problems in the improving sleep trajectory (eFigure 3B and eTable 3 in Supplement 1). When applying the same model to cortical thickness and volume (the other 2 outcomes with group differences and associations with sleep), the only substantial between-trajectory or within-trajectory difference in mTBI was between the improving and normal trajectory, which was present both before and after the injury (eTable 4 in Supplement 1).

#### Sleep Subscales

Children with mTBI mainly showed problems initiating and maintaining sleep compared with TDC (eTable 5 in Supplement 1). Of the other outcomes, only a positive association between cortical thickness and sleep breathing disorders remained after correction (eFigure 4 in Supplement 1).

### Sensitivity Analyses

When excluding children with preinjury psychiatric diagnoses, the resulting pattern differed from the main analysis: both children with mTBI and those in the OI group showed higher total sleep problems than those in the TDC group. The only remaining group difference between mTBI and controls was elevated behavioral problems compared with TDC, and behavioral problems were the only outcome associated with continuous sleep scores (eFigure 5 in Supplement 1).

When stratifying the analysis by sex, higher behavioral problems were found in girls with mTBI than in TDC, but there were no additional group differences or associations with sleep. Boys with mTBI, however, showed higher behavioral problems than TDC and lower cortical thickness compared with both control groups, as well as lower cortical volume than children with OI. Their sleep problems were associated with behavioral problems and cortical volume (eFigure 6 and eFigure 7 in Supplement 1).

No moderating effects were found between group membership and baseline sleep problems (eFigure 8 in Supplement 1). When controlling for group, baseline sleep problems in the entire sample were associated with FA (eFigure 9 in Supplement 1) and follow-up sleep problems were associated with behavior problems (eFigure 10 in Supplement 1).

## Discussion

In this cohort study, we investigated the prevalence and outcomes of sleep disturbances after pediatric mTBI, compared with both TDC and OI controls, while accounting for preinjury characteristics. Children with mTBI exhibited significantly more sleep problems than TDC, but not OI controls, suggesting that sleep disturbances may occur after an injury, rather than being specific to brain trauma alone. Sleep problems, especially newly developing ones, were consistently associated with increased behavioral problems.

Children with mTBI were more likely to develop new clinical sleep disturbances than controls (15.2% vs 11.5% in TDC and 9.9% in OI), and more frequently exhibited chronic sleep problems (21.5% vs 13.1% in both control groups). These findings indicate that children with mTBI are at risk for developing new sleep problems after the injury, but it is important to take into account highly prevalent preexisting problems, which may otherwise confound results. The longitudinal design of this study allowed us to reduce this bias by accounting for preinjury scores. Group differences in total sleep problems between mTBI and TDC were mainly related to problems with initiating and maintaining sleep. Sleep breathing disorders, sleep-wake transitions, disorders of arousal, excessive somnolence, and sleep hyperhidrosis were not significantly associated with mTBI.

Total sleep problems did not differ significantly between the mTBI and OI groups, but also not between control groups. This suggests that sleep disturbances may follow injury in general, but they may be more severe or more persistent after brain injury. This aligns with prior findings showing mixed results for OI controls,^16,17,18^ likely reflecting a more transient nature or lower severity of sleep disturbances in OI.

Children with mTBI exhibited substantially more behavioral problems than TDC. Total sleep problems were also substantially associated with higher behavioral issues in the patient cohort. Because all outcomes were measured at the same time point, we aimed to disentangle the temporal dynamics of this association by splitting patients into clinical trajectories. At baseline, children with chronic sleep problems reported significantly more behavioral difficulties than those with new-onset or no sleep issues, indicating that sleep problems were already related to behavioral issues before an injury. At the follow-up after the injury, children with new-onset sleep problems showed significantly increased behavior problems, while no other trajectory showed significant change over the 2 years and through the injury. Postinjury, both the children with chronic and new-onset sleep problems had higher behavioral issues than those with persistently normal sleep. In TDC without an injury, however, the new-onset sleep trajectory did not show these worsening behavioral problems. Instead, children with improving sleep reported an average decrease in behavioral problems over time. These findings indicate that new sleep disturbances specifically may be risk factors for the development of postinjury behavioral dysregulation and a promising target for their prevention. Clinically, this could be implemented by screening children for preexisting sleep problems at the acute stage after the injury and advising their parents to watch out for any newly occurring disturbances. If any are detected, families could then be referred to follow a stepwise approach, starting with education on sleep hygiene, then behavioral interventions, and finally medication, as well as structural accommodations, such as delayed school start times.

Changes in cognition were not impacted by mTBI or sleep. This is consistent with studies in adults, which most often find associations between sleep and psychiatric symptoms,^8,37^ rather than cognitive performance. Other research^38,39^ suggests that behavioral symptoms are influenced by both subacute and persistent sleep problems, while only subacute problems are associated with cognitive ability. Given the 2-year interval of this study, potential transient impacts of cognition may have resolved already.

Children with an mTBI had lower cortical thickness and volume compared with OI controls, but not TDC. Altered cortical thinning after pediatric moderate-to-severe TBI has been shown to be associated with behavioral and emotional dysregulation,^40^ while other studies reported higher cortical thickness after mTBI^41,42^ in association with higher postconcussive symptoms.^41^ Since we controlled for baseline values, it is unlikely that this finding was due to preinjury differences in thickness and volume. However, it is possible that there were other preinjury characteristics within the OI group that contribute to delayed maturation compared with the mTBI or TDC group, which would then become detectable after the 2-year time period of this analysis. In future ABCD releases, this question could be addressed by including the 4-year follow-up MRI scans, when normative trajectories of healthy cortical thickening and thinning during childhood development could be modeled. Despite relatively small associations of both brain structural measures with total sleep problems, the analysis of sleep trajectories did not show meaningful impacts over time. Exploratory analyses revealed an association of thickness and sleep breathing disorders specifically, indicating that the association might not be based on sleep itself, but rather on other physiological processes.

White matter microstructure (measured via FA) was unaffected by both mTBI and sleep, in line with prior studies on the ABCD data and on healthy populations.^43,44^ The injuries captured here are likely too mild to cause detectable microstructural changes up to 2 years later. Additionally, future studies using objective sleep measures or self-report, rather than parent report, may better capture these relationships.

### Limitations

Some limitations need to be discussed. First, this study relied on commonly used parent-reported measures of sleep, which may be less sensitive to subtle mTBI-related changes compared with objective measures like polysomnography or actigraphy.^45^ Moreover, sleep trajectories were assigned on the basis of a previously validated SDSC cutoff. While this type of screening approach is resource efficient, it may be subject to misclassification bias compared with clinical diagnosed sleep disorders assessed by a physician. The long study interval allowed for the incorporation of preinjury data but limited our ability to distinguish between subacute and chronic sleep problems. Additionally, the observational nature of the study and the focus on only 2 time points preclude us from causal interpretations. To fully comprehend the role of sleep disturbances after mTBI, human studies with a time lag between sleep assessment and outcomes or interventional studies are needed, as well as preclinical work examining the mechanisms of sleep after mTBI. The ABCD cohort consists of predominantly healthy, US children and any injury within the mTBI group was relatively mild. As such, generalizability is limited for children with more complex injuries, more comorbidities, or from different geographical and socioeconomic backgrounds. Moreover, the number of children treated for broken bones was insufficient to achieve complete matching for study site with the OI group. Although site was used as a covariate, the higher demographic similarity between mTBI and TDC may have influenced the comparability of group differences.

## Conclusions

In this cohort study, sleep disturbances following pediatric injuries were common compared with TDC, more so after mTBI than after OI. In particular, children with newly emerging sleep problems showed a significant increase in behavioral dysregulation. These findings highlight postinjury sleep as a modifiable risk factor and suggest that early identification and targeted intervention may offer a practical opportunity to mitigate long-term socioemotional difficulties in children following mTBI.

## Notes

Supplement 1. eAppendix 1. Methodological Clarifications eFigure 1. Flowchart detailing inclusion and exclusion of participants within the ABCD dataset eTable 1. Standardized Mean Differences before and after matching eTable 2. Associations of outcomes with sleep at follow-up in the mTBI group, controlled for baseline outcome and covariates eTable 3. Between- and within-trajectory differences in behavioral problems in children with mild traumatic brain injury (mTBI) and in typically developing children (TDC) eTable 4. Between- and within-trajectory differences in cortical thickness and cortical volume in children with mild traumatic brain injury (mTBI) eTable 5. Group differences between mTBI and controls on each sleep subscale, with adjusted confidence interval eFigure 2. Group differences to mTBI in outcomes, controlled for baseline outcomes eFigure 3. Behavioral problems in the different sleep trajectories eFigure 4. Associations of Sleep Subscales with Outcomes in children with mild traumatic brain injury eAppendix 2. Sensitivity Analyses eFigure 5. Subgroup analysis in children without pre-injury psychiatric disorders eFigure 6. Subgroup analysis in girls eFigure 7. Subgroup analysis in boys eFigure 8. Interactions between group and baseline sleep problems in predicting outcomes eFigure 9. Associations of baseline sleep problems with outcomes, controlled for baseline outcome and group membership eFigure 10. Associations of follow-up sleep problems with outcomes, controlled for baseline outcome and group membership

Supplement 2. Data Sharing Statement
