# Supplementary material for: Sleep Disturbances and Cognition, Behavior, and Brain Structure in Children With mTBI
Source: JAMA Netw Open. 2026 Mar 10;9(3):e260229. doi: 10.1001/jamanetworkopen.2026.0229 (PMC12976784; doi:10.1001/jamanetworkopen.2026.0229)
Supplement: Supplement 1. — eAppendix 1. Methodological Clarifications eFigure 1. Flowchart detailing inclusion and exclusion of participants within the ABCD dataset eTable 1. Standardized Mean Differences before and after matching eTable 2. Associations of outcomes with sleep at follow-up in the mTBI group, controlled for baseline outcome and covariates eTable 3. Between- and within-trajectory differences in behavioral problems in children with mild traumatic brain injury (mTBI) and in typically developing children (TDC) eTable 4. Between- and within-trajectory differences in cortical thickness and cortical volume in children with mild traumatic brain injury (mTBI) eTable 5. Group differences between mTBI and controls on each sleep subscale, with adjusted confidence interval eFigure 2. Group differences to mTBI in outcomes, controlled for baseline outcomes eFigure 3. Behavioral problems in the different sleep trajectories eFigure 4. Associations of Sleep Subscales with Outcomes in children with mild traumatic brain injury eAppendix 2. Sensitivity Analyses eFigure 5. Subgroup analysis in children without pre-injury psychiatric disorders eFigure 6. Subgroup analysis in girls eFigure 7. Subgroup analysis in boys eFigure 8. Interactions between group and baseline sleep problems in predicting outcomes eFigure 9. Associations of baseline sleep problems with outcomes, controlled for baseline outcome and group membership eFigure 10. Associations of follow-up sleep problems with outcomes, controlled for baseline outcome and group membership [file jamanetwopen-e260229-s001.pdf]

Supplemental Online Content

Betz AK, MacLaren HR, Villagran Asiares AG, Schuhmacher LS, Koerte IK. Sleep disturbances and cognition, behavior, and brain structure in children with mTBI. *JAMA Netw Open*. 2026;9(3):e260229. doi:10.1001/jamanetworkopen.2026.0229

eAppendix 1. Methodological Clarifications**eFigure 1.** Flowchart detailing inclusion and exclusion of participants within the ABCD dataset

eTable 1. Standardized Mean Differences before and after matching

eTable 2. Associations of outcomes with sleep at follow-up in the mTBI group, controlled for baseline outcome and covariates

eTable 3. Between- and within-trajectory differences in behavioral problems in children with mild traumatic brain injury (mTBI) and in typically developing children (TDC)

eTable 4. Between- and within-trajectory differences in cortical thickness and cortical volume in children with mild traumatic brain injury (mTBI)

eTable 5. Group differences between mTBI and controls on each sleep subscale, with adjusted confidence interval

eFigure 2. Group differences to mTBI in outcomes, controlled for baseline outcomes

eFigure 3. Behavioral problems in the different sleep trajectories

eFigure 4. Associations of Sleep Subscales with Outcomes in children with mild traumatic brain injury

eAppendix 2. Sensitivity Analyses

eFigure 5. Subgroup analysis in children without pre-injury psychiatric disorders

eFigure 6. Subgroup analysis in girls

eFigure 7. Subgroup analysis in boys

eFigure 8. Interactions between group and baseline sleep problems in predicting outcomes

eFigure 9. Associations of baseline sleep problems with outcomes, controlled for baseline outcome and group membership

eFigure 10. Associations of follow-up sleep problems with outcomes, controlled for baseline outcome and group membership

This supplemental material has been provided by the authors to give readers additional information about their work.

eAppendix 1. Methodological Clarifications

1.1 mTBI Exposure

In the OTBI questionnaire, parents report whether a head-injury event occurred (e.g., “Was your child treated for a head injury due to a fall?”). If endorsed, they complete follow-up items assessing: (1) loss of consciousness, (2) memory loss, or (3) feeling dazed/confused and for how long the respective symptom lasted. The ABCD study classifies children with criteria (2) or (3) as “possible mTBI” and those with (1) as “mTBI”. Because this distinction is not typically made in clinical practice, we combined both groups into a single mTBI category.

1.2 Inclusion and Exclusion Criteria

In the mTBI group, children were included if they matched the criteria for mTBI exposure, did not sustain an mTBI before the baseline assessment, had no history of moderate-severe TBI and had complete data on the covariates (also used at matching variables).

For controls, children without neurological conditions (e.g., epilepsy) in the medical history, without mTBI at any point before the 2-year follow-up, and with complete covariates were considered. If children did not report broken bones in the two-year timeframe of this analysis, they were eligible for the typically developing children (TDC) group. If they did have broken bones within those two years, they were eligible as orthopedic injury (OI) controls.

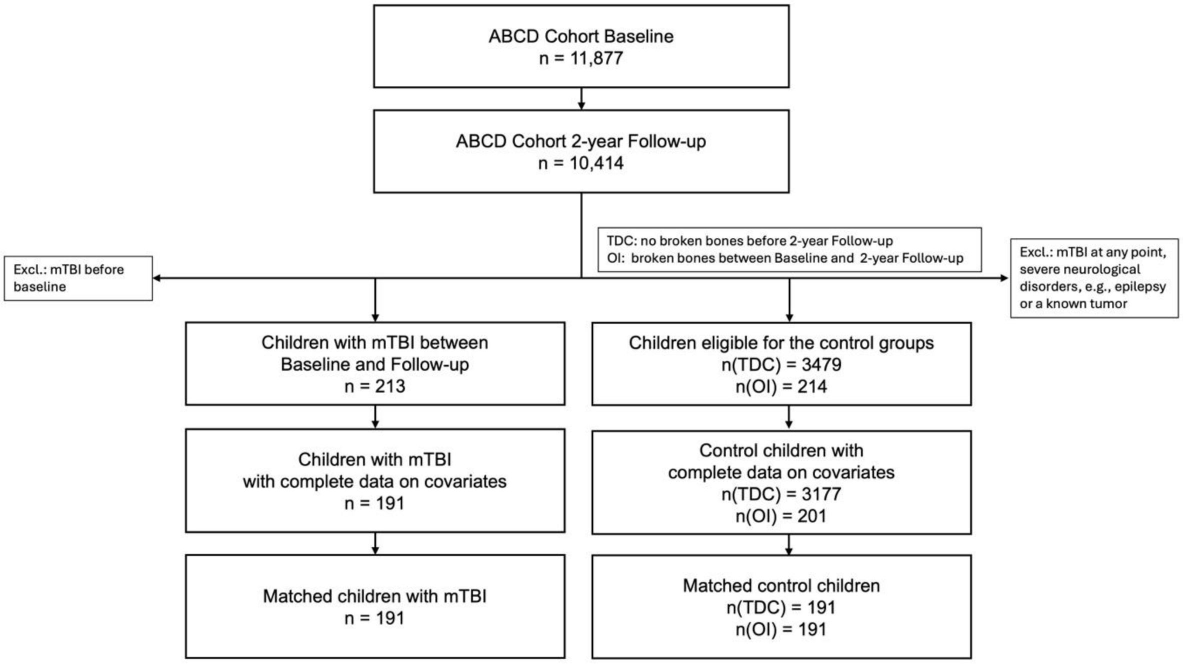

eFigure 1. Flowchart detailing inclusion and exclusion of participants within the ABCD dataset. mTBI = mild traumatic brain injury, TDC = typically developing children, OI = orthopedic injury.

© 2026 Betz AK et al. *JAMA Network Open*.

1.3 Standardized Mean Differences between mTBI and Control Groups

	mTBI-TDC before Matching	mTBI-TDC after Matching	mTBI-OI before Matching	mTBI-OI after Matching
distance	-0.5039	-0.01463	-0.87851	-0.77168
interview_age	0.107098	-0.03185	0.088931	0.050154
sexF	-0.20194	-0.05316	-0.11985	-0.11694
sexM	0.201937	0.053155	0.119851	0.116942
demo_comb_income_v21	0.023987	-0.06558	0.132797	0.131165
demo_comb_income_v22	-0.16642	0	-0.22168	0
demo_comb_income_v23	-0.11668	-0.10287	-0.04376	-0.05143
demo_comb_income_v24	-0.04698	0.032791	0.008157	0
demo_comb_income_v25	-0.07633	0	-0.09621	-0.05573
demo_comb_income_v26	-0.01738	-0.15104	0.033385	0.021577
demo_comb_income_v27	-0.06514	0.164001	0.064703	0.0656
demo_comb_income_v28	-0.08622	0.031591	-0.05619	-0.07898
demo_comb_income_v29	0.036154	0	-0.06541	-0.10866
demo_comb_income_v210	0.217439	-0.02487	0.109928	0.111907
site_id_lsite01	-0.0274	0.036564	-0.27068	-0.25595
site_id_lsite02	0.021575	0	-0.13719	-0.15731
site_id_lsite03	-0.42899	0.051434	-0.19038	-0.20574
site_id_lsite04	0.069385	0.089601	0.237419	0.250882
site_id_lsite05	-0.34616	-0.07255	-0.61684	-0.29019
site_id_lsite06	0.037788	0.044947	0.033654	0.022473
site_id_lsite07	0.157015	-0.02471	0.151938	0.14825
site_id_lsite08	0.041144	0	0.037481	0.030015
site_id_lsite09	0.141822	-0.11237	-0.00906	-0.02247
site_id_lsite10	-0.09106	-0.09883	-0.12981	-0.12354
site_id_lsite11	-0.01641	-0.03656	0.042021	0.036564
site_id_lsite12	0.035906	0.055728	0.062659	0.055728
site_id_lsite13	-0.12245	0.026135	0.134578	0.130677
site_id_lsite14	-0.12491	0.083592	-0.09621	-0.11146
site_id_lsite15	0.037582	0.055728	0.142092	0.167183
site_id_lsite16	0.05811	0.0656	0.002366	-0.0164
site_id_lsite17	0.118315	0.056695	0.050959	0.037796
site_id_lsite18	-0.03104	-0.03002	0.037481	0.06003
site_id_lsite19	-0.11821	-0.05573	-0.06973	-0.08359
site_id_lsite20	-0.14453	-0.04942	-0.20025	-0.22237
site_id_lsite21	0.100136	-0.13229	0.104833	0.094491
demo_race_adaptedAIAN	0.093413	0.084215	0.126323	0.126323
demo_race_adaptedAsian	-0.10431	0.051434	-0.04376	-0.05143
demo_race_adaptedBlack	-0.21592	0	0.052953	0.083153
demo_race_adaptedMultiple	0.098621	0.014389	0.117187	0.115111
demo_race_adaptedNHPI	-0.03165	0	0	0
demo_race_adaptedOther	-0.05487	0	0.039317	0.032791
demo_race_adaptedWhite	0.060754	-0.04677	-0.16417	-0.17539

eTable 1. Standardized Mean Differences before and after matching.

© 2026 Betz AK et al. *JAMA Network Open*.

1.4 Selection of Outcome Measures, Standardization, Multiple Comparisons

This study focusses on high-level outcome measures (e.g., total scores, whole-brain metrics). To maintain a consistent level of analysis across domains, we did not include any subscales or regional brain metrics. The only exception to this is the exploratory analysis of sleep subscales.

Note, that cognitive tests were selected based on availability: The ABCD study does not perform the full NIH Toolbox battery at every timepoint; although fluid, crystallized, and total composite scores are available at baseline, they are not available at the 2-year follow-up. Because previous literature does not provide strong hypotheses for specific cognitive domains, we constructed a substitute composite-score from the three age-corrected standard scores available.

The reported z-scores were computed within each bootstrap-sample. Therefore, they do not convey clinical deviation from a reference population. Because cognitive/behavioral measures (e.g., 0 – 80 points) and MRI measures (e.g., multiple 1000 mm^3 or 0-1) differ markedly in scale, standardization like z-scoring facilitates consistent visualization and comparison across outcomes.

The outcomes examined (behavior, cognition, cortical thickness, volume, fractional anisotropy) correspond to distinct hypotheses and therefore do not require global correction across all domains. Likewise, comparisons with TDC and OI are conceptually independent based on prior literature. We applied multiple-comparison adjustments where appropriate:

- Sleep subscales: six tests
  → adjusted by replacing the 0.025 quantiles with 0.004166.
- Trajectory analyses:
  - four trajectories × two timepoints (12 comparisons)
    → quantiles = 0.00208
  - baseline‑follow-up contrasts within four trajectories (4 comparisons)
    → quantiles = 0.00625

© 2026 Betz AK et al. *JAMA Network Open*.

eAppendix 2. eTables and eFigures

Outcome	Mean β	95%-CI of β	Mean f^2	95%-CI of f^2
**Cortical Thickness**	**0.13**	**[0.04, 0.22]**	**0.09**	**[0.01, 0.24]**
**Cortical Volume**	**0.05**	**[0.01, 0.09]**	**0.07**	**[0.00, 0.20]**
Fractional Anisotropy	-0.02	[-0.23, 0.17]	0.01	[0.00, 0.07]
Cognition	-0.03	[-0.17, 0.12]	0.01	[0.00, 0.07]
**Behavior Problems**	**0.33**	**[0.13, 0.52]**	**0.18**	**[0.02, 0.41]**

eTable 2. Associations of outcomes with sleep at follow-up in the mTBI group, controlled for baseline outcome and covariates. Bold text shows a confidence interval that does not include 0, indicative of statistical significance.

Timepoint	Comparison	Mean β mTBI	95% CI mTBI	Mean β TDC	95% CI TDC
2-year Follow-up	Chronic-Improving	0.87	[-0.19, 1.78]	0.62	[-0.23, 1.48]
	Chronic-New Onset	-0.07	[-0.99, 0.92]	0.18	[-0.64, 1.01]
	**Chronic-Normal**	**1.01**	**[0.32, 1.71]**	**1.16**	**[0.45, 1.83]**
	Improving-New Onset	-0.94	[-2.01, 0.08]	-0.44	[-1.16, 0.26]
	Improving-Normal	0.14	[-0.67, 0.98]	0.54	[-0.03, 1.08]
	**New Onset-Normal**	**1.08**	**[0.32, 2.00]**	**0.98**	**[0.48, 1.53]**
Baseline	Chronic-Improving	0.99	[-0.19, 1.97]	0.62	[-0.40, 1.55]
	**Chronic-New Onset**	**1.07**	**[0.33, 1.89]**	0.81	[-0.06, 1.73]
	**Chronic-Normal**	**1.22**	**[0.51, 1.91]**	**1.52**	**[0.54, 2.34]**

© 2026 Betz AK et al. *JAMA Network Open*.

Timepoint	Comparison	Mean β mTBI	95% CI mTBI	Mean β TDC	95% CI TDC
	Improving-New Onset	0.08	[-1.00, 1.23]	0.19	[-0.50, 0.91]
	**Improving-Normal**	0.22	[-0.84, 1.34]	**0.91**	**[0.37, 1.50]**
	**New Onset-Normal**	0.14	[-0.49, 0.84]	**0.71**	**[0.12, 1.33]**
Change over time (2-year – Baseline)	Chronic	-0.24	[-0.68, 0.18]	-0.47	[-0.99, 0.03]
	**Improving**	-0.12	[-0.52, 0.30]	**-0.47**	**[-0.79, -0.17]**
	**New Onset**	**0.90**	**[0.48, 1.40]**	0.16	[-0.39, 0.66]
	Normal	-0.03	[-0.22, 0.16]	-0.11	[-0.25, 0.02]

eTable 3. Between- and within-trajectory differences in behavioral problems in children with mild traumatic brain injury (mTBI) and in typically developing children (TDC). Bold text shows a confidence interval in one or both groups that does not include 0, indicative of statistical significance.

Outcome	Timepoint	Comparison	Mean β	95% CI
Thickness	2-year Follow-up	Chronic-Improving	0.20	[-0.93, 1.13]
		Chronic-New Onset	0.29	[-0.79, 1.37]
		Chronic-Normal	-0.19	[-0.96, 0.47]
		Improving-New Onset	0.09	[-1.16, 1.19]
		Improving-Normal	-0.38	[-1.33, 0.53]
		New Onset-Normal	-0.48	[-1.48, 0.62]
	Baseline	Chronic-Improving	-0.10	[-1.11, 0.87]
		Chronic-New Onset	0.29	[-0.85, 1.32]
		Chronic-Normal	-0.36	[-1.08, 0.30]
		Improving-New Onset	0.39	[-0.86, 1.68]
		Improving-Normal	-0.27	[-1.13, 0.73]
		New Onset-Normal	-0.65	[-1.63, 0.43]

© 2026 Betz AK et al. *JAMA Network Open*.

Outcome	Timepoint	Comparison	Mean β	95% CI
	Change over time (2-year – Baseline)	Chronic	0.03	[-0.15, 0.18]
		Improving	-0.27	[-0.60, 0.03]
		New Onset	0.03	[-0.33, 0.32]
		Normal	-0.15	[-0.30, 0.01]
Volume	2-year Follow-up	Chronic-Improving	0.66	[-0.17, 1.51]
		Chronic-New Onset	0.03	[-0.53, 0.73]
		Chronic-Normal	-0.17	[-0.76, 0.31]
		Improving-New Onset	-0.63	[-1.51, 0.38]
		**Improving-Normal**	**-0.83**	**[-1.63, -0.09]**
		New Onset-Normal	-0.20	[-0.82, 0.42]
	Baseline	Chronic-Improving	0.61	[-0.17, 1.47]
		Chronic-New Onset	0.05	[-0.55, 0.83]
		Chronic-Normal	-0.21	[-0.75, 0.25]
		Improving-New Onset	-0.56	[-1.37, 0.35]
		**Improving-Normal**	**-0.83**	**[-1.62, -0.12]**
		New Onset-Normal	-0.27	[-0.90, 0.34]
	Change over time (2-year – Baseline)	Chronic	-0.01	[-0.11, 0.07]
		Improving	-0.06	[-0.17, 0.08]
		New Onset	0.01	[-0.10, 0.10]
		Normal	-0.06	[-0.11, 0.00]

eTable 4. Between- and within-trajectory differences in cortical thickness and cortical volume in children with mild traumatic brain injury (mTBI). Bold text shows a confidence interval in one or both groups that does not include 0, indicative of statistical significance.

© 2026 Betz AK et al. *JAMA Network Open*.

Outcome	Group	Mean β	95%-CI of β	Mean f^2	95%-CI of f^2
**DIMS**	**TDC**	**-0.23**	**[-0.50, -0.02]**	**0.01**	**[0.00, 0.06]**
	OI	-0.11	[-0.38, 0.14]	0.01	[0.00, 0.03]
SBD	TDC	-0.13	[-0.35, 0.10]	0.01	[0.00, 0.03]
	OI	-0.02	[-0.22, 0.23]	0.00	[0.00, 0.01]
DA	TDC	-0.10	[-0.35, 0.18]	0.00	[0.00, 0.03]
	OI	-0.03	[-0.27, 0.25]	0.00	[0.00, 0.02]
SWT	TDC	-0.22	[-0.45, 0.03]	0.02	[0.00, 0.06]
	OI	-0.06	[-0.24, 0.17]	0.00	[0.00, 0.02]
DOES	TDC	-0.15	[-0.38, 0.07]	0.01	[0.00, 0.04]
	OI	-0.16	[-0.40, 0.05]	0.01	[0.00, 0.04]
SHY	TDC	-0.10	[-0.33, 0.14]	0.01	[0.00, 0.03]
	OI	-0.02	[-0.23, 0.18]	0.00	[0.00, 0.01]

eTable 5. Group differences between mTBI and controls on each sleep subscale, with adjusted confidence interval. mTBI = mild traumatic brain injury, TDC = typically developing children, OI = orthopedic injury, DIMS = Disorders of Initiating and Maintaining Sleep, SBD = Sleep Breathing Disorders, DA = Disorder of Arousal, SWT = Sleep-Wake Transition Disorders, DOES = Disorders of Excessive Somnolence, SHY = Sleep Hyperhidrosis.

© 2026 Betz AK et al. *JAMA Network Open*.

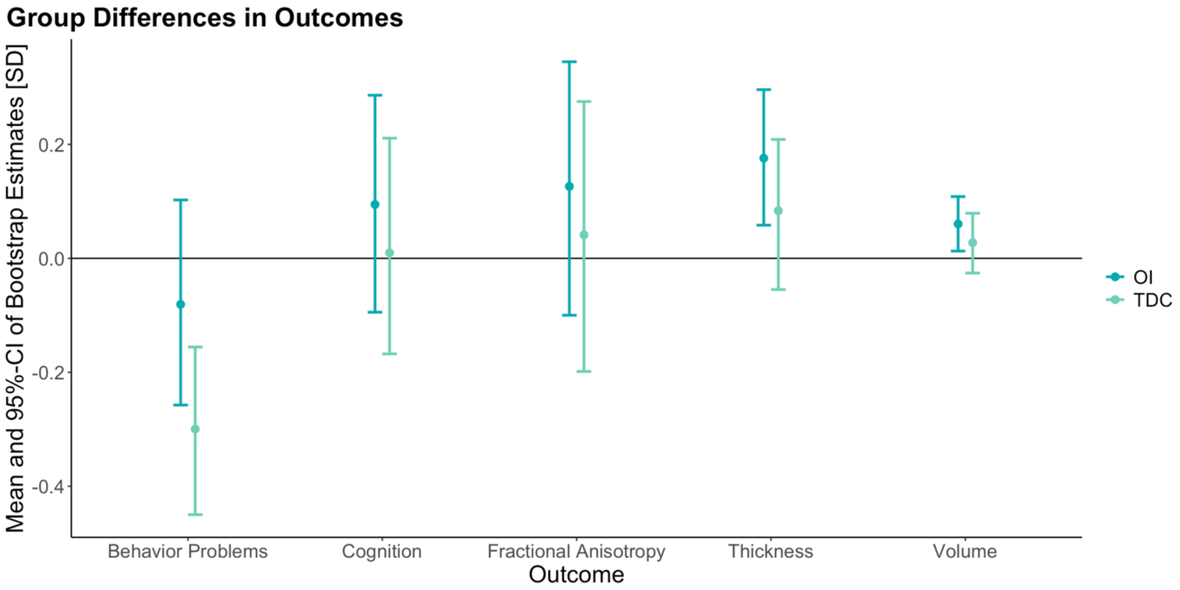

eFigure 2. Group differences to mTBI in outcomes, controlled for baseline outcomes. TDC = typically developing children, OI = orthopedic injury.

© 2026 Betz AK et al. *JAMA Network Open*.

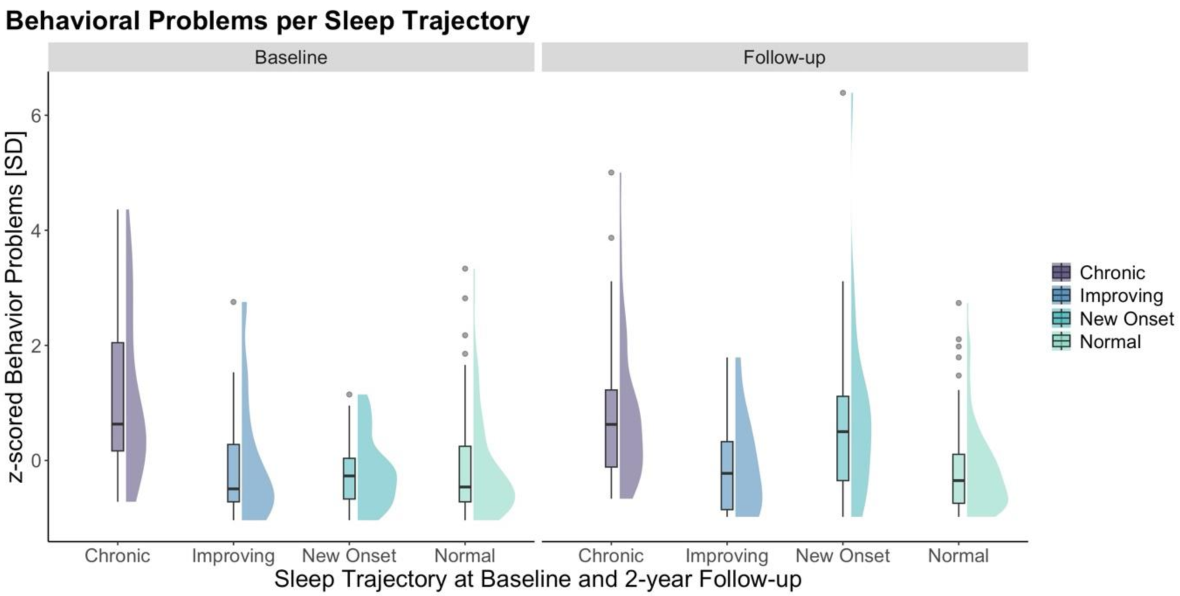

(A)

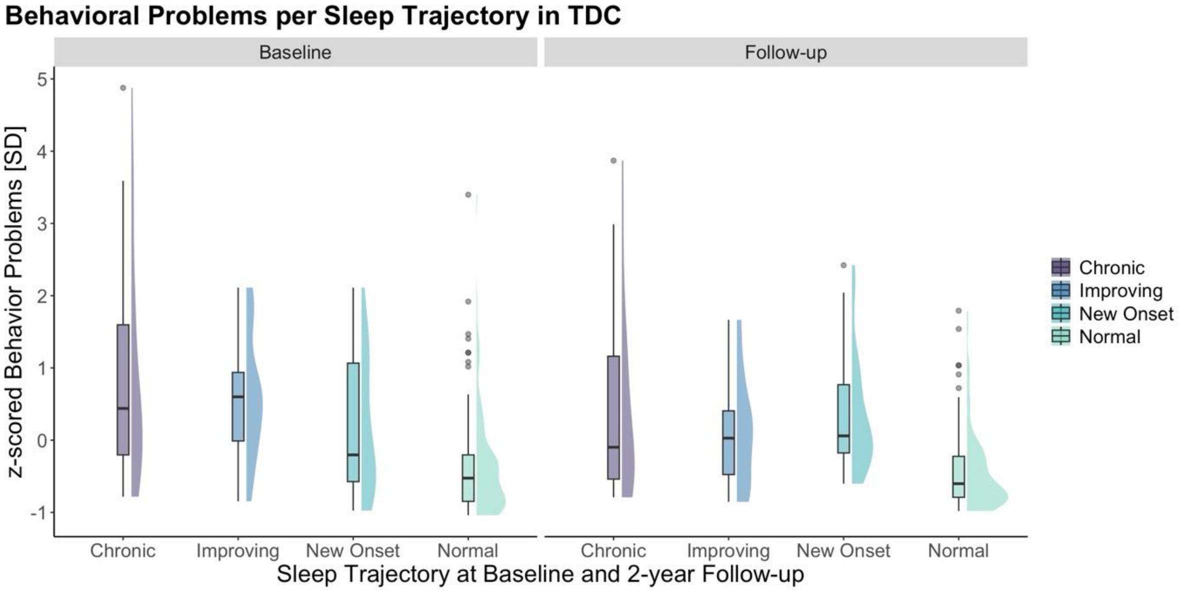

(B)

eFigure 3. Behavioral problems in the different sleep trajectories. In children with mild traumatic brain injury (A), this represents before (baseline) and after (follow-up) the injury. In typically developing children (TDC, (B)), this represents the development over two years without a disrupting event.

© 2026 Betz AK et al. *JAMA Network Open*.

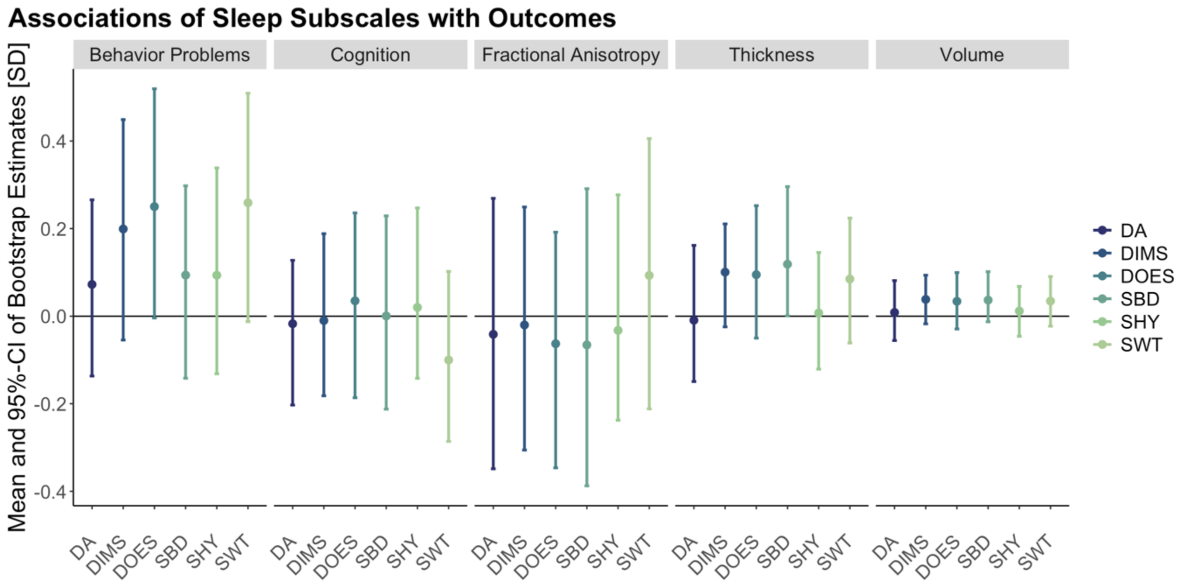

eFigure 4. Associations of Sleep Subscales with Outcomes in children with mild traumatic brain injury. DA = Disorder of Arousal, DIMS = Disorders of Initiating and Maintaining Sleep, DOES = Disorders of Excessive Somnolence, SBD = Sleep Breathing Disorders, SHY = Sleep Hyperhidrosis, SWT = Sleep-Wake Transition Disorders.

© 2026 Betz AK et al. *JAMA Network Open*.

eAppendix 3. Sensitivity Analyses

3.1 Pre-Injury Psychiatric Vulnerability

Children were excluded from the sample based on the KSADS diagnostic parent interview, if any of the diagnoses were *present* at the baseline timepoint. *Past* diagnoses or those *in remission / partial remission* were not excluded for this analysis. The eligible mTBI participants were matched to new controls, where children with psychiatric diagnoses at baseline had also been excluded from the pool.

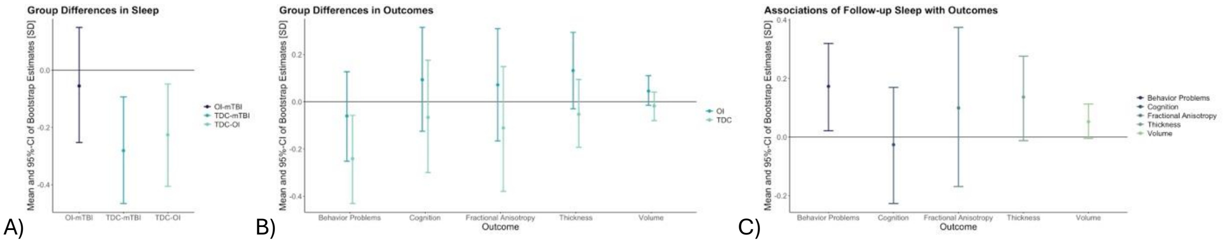

eFigure 5. Subgroup analysis in children without pre-injury psychiatric disorders. Group differences in follow-up/post-injury sleep problems between mild traumatic brain injury (mTBI), orthopedic injury (OI), and typically developing children (TDC) (A). Group differences in outcomes between mTBI and OI/TDC (B). Associations of sleep with outcomes in the mTBI group (C).

3.2 Sex-Stratified Analyses

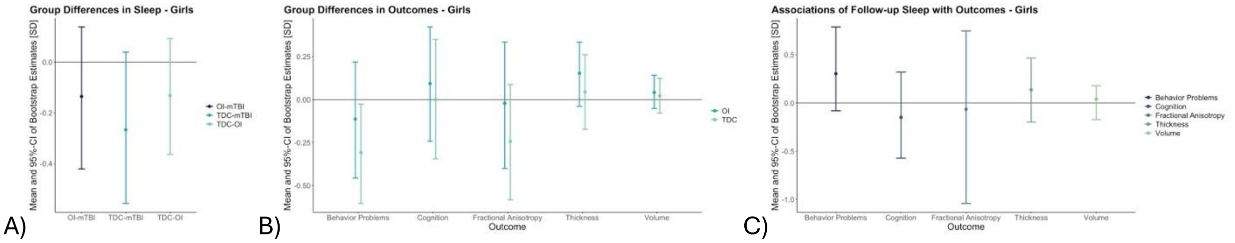

eFigure 6. Subgroup analysis in girls. Group differences in follow-up/post-injury sleep problems between mild traumatic brain injury (mTBI), orthopedic injury (OI), and typically developing children (TDC) (A). Group differences in outcomes between mTBI and OI/TDC (B). Associations of sleep with outcomes in the mTBI group (C).

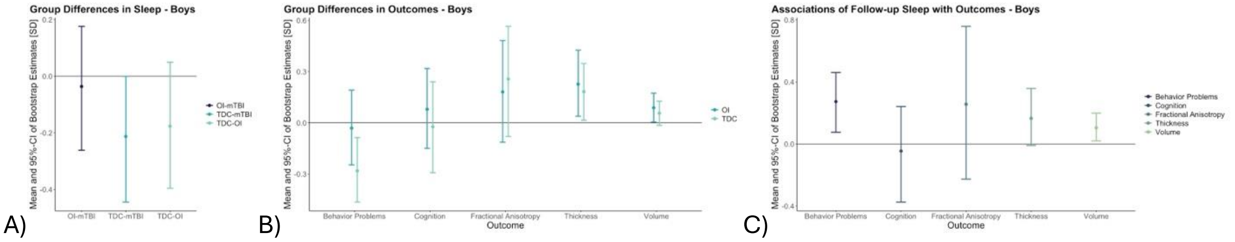

eFigure 7. Subgroup analysis in boys. Group differences in follow-up/post-injury sleep problems between mild traumatic brain injury (mTBI), orthopedic injury (OI), and typically developing children (TDC) (A). Group differences in outcomes between mTBI and OI/TDC (B). Associations of sleep with outcomes in the mTBI group (C).

3.3 The Role of Pre-Injury Sleep

Based on recent literature, we additionally examined whether group and pre-injury sleep disturbances interact to predict other outcomes. However, because no

© 2026 Betz AK et al. *JAMA Network Open*.

moderating effects were found, we also show main effects of baseline and follow-up sleep controlling for group.

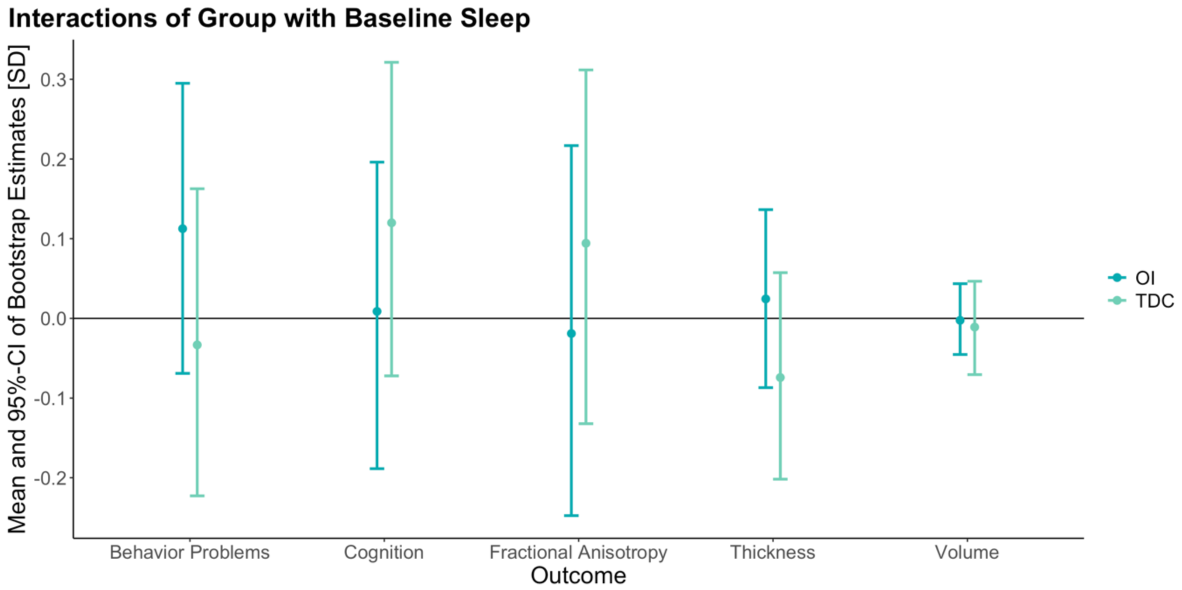

eFigure 8. Interactions between group and baseline sleep problems in predicting outcomes. TDC = typically developing children, OI = orthopedic injury.

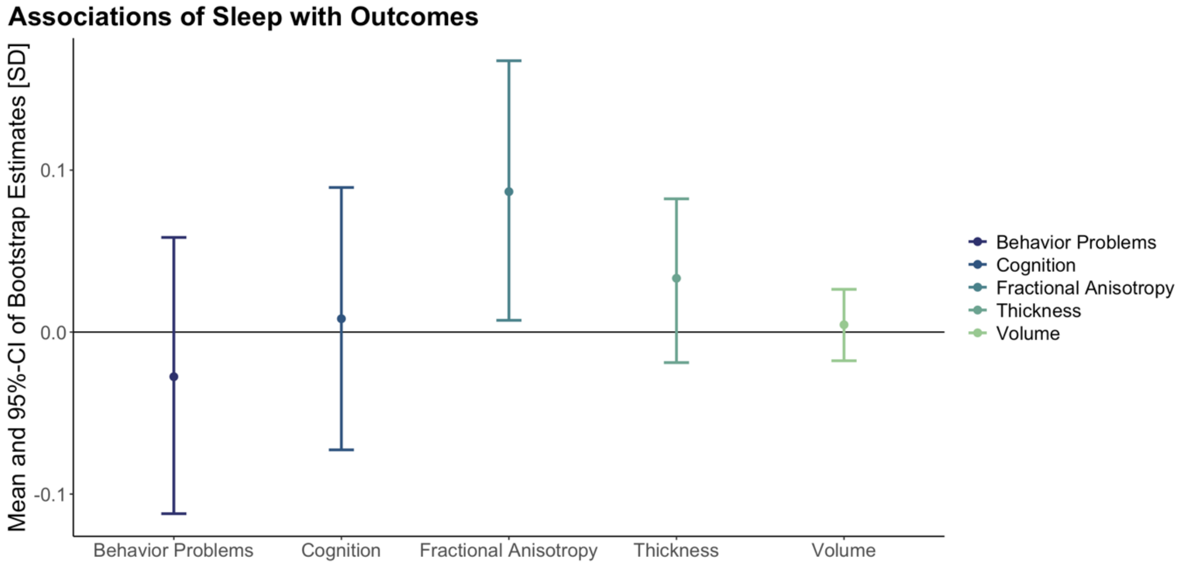

eFigure 9. Associations of baseline sleep problems with outcomes, controlled for baseline outcome and group membership.

© 2026 Betz AK et al. *JAMA Network Open*.

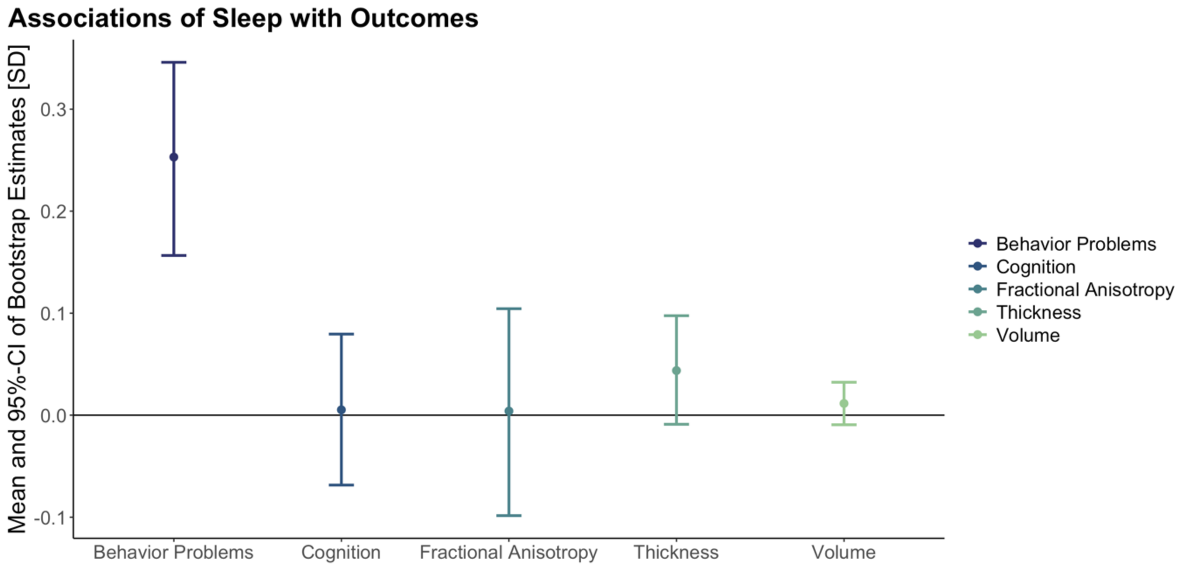

eFigure 10. Associations of follow-up sleep problems with outcomes, controlled for baseline outcome and group membership.

© 2026 Betz AK et al. *JAMA Network Open*.
