# Supplementary material for: Sleep Disturbances and Cognition, Behavior, and Brain Structure in Children With mTBI
Source: JAMA Netw Open. 2026 Mar 10;9(3):e260229. doi: 10.1001/jamanetworkopen.2026.0229 (PMC12976784; doi:10.1001/jamanetworkopen.2026.0229)
Supplement: Supplement 2. — Data Sharing Statement [file jamanetwopen-e260229-s002.pdf]

Data Sharing Statement

Betz. Sleep Disturbances and Cognition, Behavior, and Brain Structure in Children With mTBI. *JAMA Netw Open*. Published March 10, 2026. doi:10.1001/jamanetworkopen.2026.0229

Data
Data available: No

Additional Information
Explanation for why data not available: Data cannot be shared by the study team since this is a secondary data analysis. The curated, anonymized ABCD dataset is released annually to any researcher holding a valid data use agreement. The analysis code that can be reproduced with the ABCD tabulated data has been released with a Zenodo DOI (https://zenodo.org/records/18301365). AKB and IKK had full access to all the data in the study and takes responsibility for the integrity of the data and the accuracy of the data analysis.
